# Deep learning-based prediction of cerebral white matter hyperintensity burden using carotid magnetic resonance angiography

**DOI:** 10.3389/fneur.2025.1656705

**Published:** 2025-09-09

**Authors:** Jiho Lee, Kyu Sung Choi, Seung Hong Choi, Inpyeong Hwang, Taehoon Shin

**Affiliations:** ^1^Department of Artificial Intelligence and Software, Ewha Womans University, Seoul, Republic of Korea; ^2^Department of Radiology, Seoul National University Hospital, Seoul National University College of Medicine, Seoul, Republic of Korea; ^3^Healthcare AI Research Institute, Seoul National University Hospital, Seoul, Republic of Korea; ^4^School of Transdisciplinary Innovations, Seoul National University, Seoul, Republic of Korea; ^5^Department of Mechanical and Biomedical Engineering, Ewha Womans University, Seoul, Republic of Korea

**Keywords:** cerebral small vessel disease, white matter hyperintensity, carotid artery, MRA, deep learning

## Abstract

**Purpose:**

White matter hyperintensities (WMHs) are key neuroimaging markers of cerebral small vessel disease (cSVD), associated with cognitive decline and increased stroke risk. We aimed to investigate whether carotid time-of-flight (TOF) magnetic resonance angiography (MRA), a routinely acquired and non-invasive vascular imaging modality, can be utilized to independently predict WMH burden using deep learning.

**Methods:**

We developed a deep learning-based framework to predict WMH presence and severity using only 3D carotid TOF MRA. Two classification tasks were defined: binary (grade 0 vs. grades 1–3) and three-class (grade 0, 1, 2–3) classification. Four model architectures— simple fully convolutional network (SFCN), ResNet10, MedicalNet, and Medical Slice Transformer—were evaluated. To enhance model interpretability, we performed saliency mapping and occlusion analysis.

**Results:**

SFCN performed the best, achieving an accuracy of 76.5% and an area under the receiver operating characteristic curve (AUC) of 0.874 in binary classification, along with a 63.5% accuracy and a 0.827 AUC in WMH severity classification. Interpretability analyses confirmed that models predominantly focused on carotid vessel regions, which supports known vascular associations with WMH burden.

**Conclusion:**

Carotid TOF MRA alone can serve as a predictive marker for WMH burden when analyzed using deep learning. This approach highlights the potential utility of extracranial carotid imaging as a non-invasive surrogate for early and accessible assessment of cerebrovascular risk.

## 1 Introduction

Cerebral small vessel disease (cSVD), an umbrella term for brain lesions attributed to small cerebral vessels, is a major contributor to stroke and dementia, in addition to being associated with neurobehavioral symptoms or functional impairment (1, 2). cSVD is characterized by several neuroimaging markers including lacunes, cerebral microbleeds and white matter hyperintensities (WMHs) (3). Among them, WMHs, appearing as high signal intensities in periventricular and deep subcortical white matter on T2-weighted – and preferably with fluid attenuated inversion recovery (FLAIR) – magnetic resonance imaging (MRI), represent the most commonly encountered radiological features in cSVD (4). WMH burden, typically assessed using visual rating scales, is correlated with neurological outcomes, including cognitive impairment, gait disturbances, and an increased risk of stroke and dementia (5–7). Early detection and quantification of WMHs may help identify individuals at risk and contribute to preventing or delaying the progression of cSVD.

The clinical significance of WMHs has prompted investigations into alternative screening approaches beyond conventional brain MRI. Fundus photography has been proposed as a surrogate approach due to the anatomical and physiological parallels between retinal and cerebral microvasculature (8). Studies have progressed from utilizing automatic retinal image analysis with machine learning (9, 10) to employing convolutional neural networks (CNNs) for direct WMH severity classification (here referring to the semi-quantitative ordinal grading of WMH burden) in an end-to-end manner (11, 12). These efforts underscore a growing interest in using extracranial imaging — particularly from anatomically distinct but physiologically related systems — to develop accessible and cost-effective tools for WMH screening.

The cervical internal carotid artery, which can be assessed through MR angiography (MRA) as well as more accessible modalities, such as ultrasound or computed tomography (CT) angiography, has drawn attention as an extracranial vasculature linked to WMH burden. Since the internal carotid arteries supply up to 75% of the cerebral blood flow, their structural and hemodynamic characteristics are critical for cerebral perfusion (13). Several studies have reported associations between carotid abnormalities and WMH burden, including larger carotid diameters and increased carotid intima-media thickness being linked to greater WMH burden (14, 15). Morphological features such as increased tortuosity have also been proposed as independent predictors of WMH severity in patients with acute ischemic stroke (16), while advanced multimodal imaging techniques including MRA, ultrasound, and 4D flow MRI have revealed potential links between altered flow patterns and cSVD progression (17). However, research thus far has focused predominantly on statistical associations rather than predictive modeling. To the best of our knowledge, WMH severity has yet to be directly predicted based on carotid MRA alone, while deep learning applications also remain unexplored in this context.

The purpose of this study was to develop and evaluate a deep learning-based framework for predicting the presence and severity of WMH using clinical carotid time-of-flight (TOF) MRA images. Specifically, we focused on binary classification (absence vs. presence of WMH) and three-class severity classification (Fazekas grade 0, grade 1, and grades 2–3) for WMH. We evaluated both convolutional neural networks, e.g., simple fully convolutional networks (SFCNs) and ResNet variants, along with a transformer-based model, i.e., Medical Slice Transformer (MST), while analyzing the spatial contributions of vascular structures to enhance model interpretability. This approach represents a non-invasive and generalizable strategy for WMH screening and may facilitate the early detection of cerebrovascular changes in individuals at risk of cSVD.

## 2 Materials and methods

### 2.1 Data acquisition and WMH grading

This study utilized non-contrast brain MRI and carotid 3D TOF MRA data acquired as part of a comprehensive health check-up program at Seoul National University Hospital, between 2016 and 2023. All images were obtained using a 3.0 Tesla MR scanner (Discovery 750w, GE Healthcare, Milwaukee, WI) with a head and neck coil. To focus on vascular features without major cerebral pathology, patients with overt brain lesions, significant stenosis in extra-cranial or intra-cranial cerebral arteries, or clinically diagnosed cerebrovascular disease were excluded based on imaging reports and medical records. Accordingly, a total of 1,105 patients were included in the final dataset, comprising 504 females and 601 males. The ages ranged from 20 to 91 years, with the mean being 63.9 ± 11.1 years. The brain MRI included fast spin-echo axial T2-weighted FLAIR images with the following parameters: repetition time (TR), 8,000 ms; echo time (TE), 90.7 ms; inversion time, 2,347 ms; echo train length, 26; flip angle (FA), 142°; field-of-view (FOV), 220 mm × 220 mm; acquisition matrix, 288 × 288; slice thickness, 5 mm; interslice gap, 1 mm; and number of excitations, 1. Three-dimensional carotid TOF MRA were obtained with the following parameters: TR, 18 ms; TE, 3.4 ms; FA, 18°; FOV, 300 mm × 300 mm; acquisition matrix, 320 × 192; reconstruction matrix (applying zero-interpolation filling), 512 × 512; slice thickness/slice interval 2 mm/1 mm; and multiple overlapping thin slab acquisition (MOTSA) with 4 slabs. The reconstructed voxel resolution was 0.586 × 0.586 × 1.0 mm.

Based on the FLAIR images, WMH severity was visually graded by two investigator (I. H. and K. S. C, with 13 and 11 years of experience in neuroradiology) on a modified Fazekas scale (18, 19), a four-point scale (0–3) where 0 indicates no WMHs, including symmetrical, well-defined caps/bands or occasional punctate lesions; 1 represents multiple punctate foci; 2 indicates the beginning confluence of lesions; and 3 reflects large confluent areas. The inter-observer reliability was evaluated using weighted kappa, yielding a value of 0.879 (95% CI: 0.863–0.895), indicating almost perfect agreement. Any discrepancies were resolved by the consensus. The grade distribution within the dataset was as follows: grade 0, 461 patients; grade 1, 372 patients; grade 2, 194 patients; and grade 3, 77 patients.

### 2.2 Data preprocessing

All TOF MRA volumes were converted from DICOM to NIfTI format to facilitate downstream processing. Each volume was resampled to a voxel spacing of 1.0 × 1.0 × 1.0 mm using linear interpolation with SimpleITK (20), to ensure consistent spatial resolution across subjects and enable uniform feature representation for model training. The resulting in-plane dimensions were 300 × 300 voxels, with the number of axial slices varying from 192 to 272 across subjects. The carotid bifurcation was manually identified on maximum intensity projection (MIP) images, and each volume was cropped to a fixed size of 112 × 84 × 112 voxels (superior–inferior × anterior–posterior × right–left) centered at the bifurcation. This size was empirically found to exclude non-informative background regions while including all the relevant arteries. The voxel intensities were then normalized on a per-subject basis via z-score normalization, to reduces inter-subject intensity variability and improve training stability.

### 2.3 Deep learning networks

**Figure 1:**
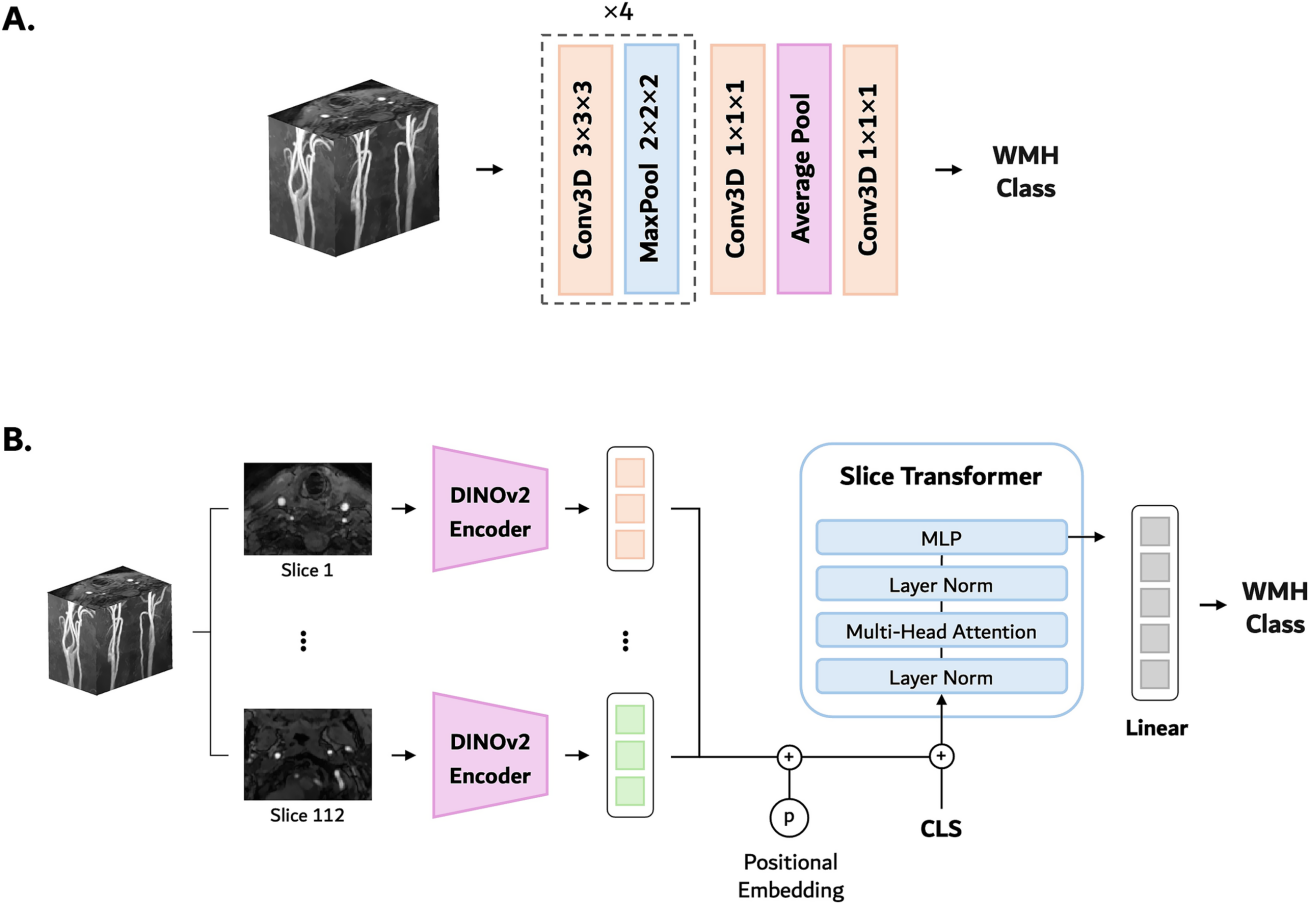
Deep learning network architectures used for WMH classification: **(A)** SFCN, **(B)** MST.

We implemented and compared several deep learning models for WMH prediction, including 3D CNNs and a transformer-based model. Carotid TOF MRA is characterized by sparse vascular signal, low structural complexity, and large background regions of low signal. These conditions along with limited training samples encouraged us to select lightweight CNNs as over-parameterized networks are often unnecessary and prone to overfitting (21, 22). We also chose a transformer-based model to enable learning of long-range dependencies between distance slices in 3D MRA data. Figure 1 presents an overview of the model architectures used.

#### 2.3.1 Convolutional networks

We employed three 3D CNN architectures: a standard ResNet (23), a ResNet variant initialized with pretrained weights from MedicalNet (24), and an SFCN (22). ResNet10, the lightest variant of a commonly used CNN backbone, was adopted for its simplicity and proven effectiveness across numerous image analysis applications. We also evaluated the MedicalNet model, which was based on the ResNet10 architecture and pretrained on diverse public 3D medical image datasets (3DSeg-8) covering multiple organs (brain, heart, prostate, etc.) and imaging modalities (MRI and CT). We initialized the model with pretrained weights and replaced the last layer with a linear classifier to adapt it to our WMH classification task. This pretraining was expected to improve feature generalization and accelerate model convergence. SFCN is a streamlined 3D CNN architecture without fully connected layers, originally designed to predict biological age using brain MRI. Reducing the network depth and parameter count allowed SFCN to achieve competitive performance in its original brain age prediction task as well as other neuroimaging applications while maintaining high computational efficiency (25–27). In our implementation, we simplified the architecture further by reducing the number of convolutional blocks, which empirically improved the model’s performance on our dataset.

#### 2.3.2 Transformer model

In addition to CNNs, we employed a transformer-based model, namely MST (28), which leverages pretrained DINOv2 (29) features to process 3D MRA volumes as sequences of 2D slices. Specifically, we used the DINOv2-small variant, a self-supervised vision transformer (ViT) pretrained on large-scale 2D natural image datasets. In MST, each 2D axial slice was initially encoded by the DINOv2 backbone, and further finetuned to adapt to the WMH classification task. The resulting slice-level embeddings were then processed through a transformer encoder, which incorporated a learnable class token and slice position embeddings to capture inter-slice dependencies. To obtain the final WMH grade prediction, a linear classification head was applied to the updated class token. Through this hybrid attention mechanism, MST integrates both global (inter-slice) and local (intra-slice) contextual information. Whereas 3D CNNs are well suited for capturing local spatial features in volumetric data, transformer-based architectures can more explicitly model long-range relationships across slices, which may help preserve vascular continuity in carotid TOF MRA. Its slice-wise 2D design provides a computationally efficient alternative to full 3D transformers while retaining the advantages of pretrained transformer representations.

### 2.4 Loss function

All model parameters were optimized using the cross-entropy loss function. For binary classification (i.e., WMH presence: 0 vs. 1–3), no class weighting was applied as the class distribution was relatively balanced. For the three-class classification (i.e., WMH severity: 0 vs. 1 vs. 2–3), class weights were applied to compensate for more pronounced imbalance. The weighted cross-entropy loss function is defined as follows:


$$L_{\mathrm{wCE}}=-\sum_{i=1}^{C}w_{i}\cdot y_{i}\cdot \log(\hat{y_{i}})$$

where C is the number of classes, $y_{i}$ is the ground truth label for class i , $\hat{y_{i}}$ is the predicted probability of class i , and $w_{i}$ is the weight assigned to class i . The class weights $w_{i}$ were computed based on the inverse frequency of each class and normalized as follows:


$$w_{i}=\frac{1/n_{i}}{\frac{1}{C}\sum_{j=1}^{C}1/n_{j}}$$

where $n_{i}$ represents the number of samples in class i . This normalization ensured balanced contributions from all classes to the loss, thereby maintaining a consistent loss scale.

### 2.5 Experimental setup

This study involved two classification tasks: (1) binary classification of WMH presence (grade 0 vs. grades 1–3) and (2) three-class classification of WMH severity (grade 0 vs. grade 1 vs. grades 2–3). Grades 2 and 3 were merged due to the limited number of grade 3 samples, to ensure sufficient representation and stable training. This grouping also reflects prior studies both those using retinal images as extracranial surrogates and various clinical studies, in which Fazekas grades 2 and 3 are combined into a single “moderate–severe WMH” category (9–11, 32, 33). The dataset was divided into training, validation, and test sets in an 8:1:1 ratio using stratified sampling based on a combined key of WMH grade and age group, with age binned in 10-year intervals. This ensured balanced class distributions and demographic consistency across the subsets, with all splits performed at the subject level to prevent data leakage. Experiments were repeated with five different random seeds, with each seed producing a new stratified split of the dataset into training, validation, and test sets. Model performance is reported as the mean value with 95% confidence intervals across the five runs, capturing variability due to model initialization and data partitioning. To enhance model generalizability, data augmentation was performed during training, which included random affine transformations with rotations of up to 10°, translations of up to 4 voxels, and horizontal flipping based on a probabilistic strategy. All models were trained using the AdamW optimizer, with the ReduceLROnPlateau scheduler being employed to reduce the learning rate when the validation loss plateaued. The learning rates were tuned manually separately for each model to optimize performance: 1 × 10^−5^ for ResNet10, 1 × 10^−4^ for MedicalNet, 5 × 10^−4^ for SFCN, and 1 × 10^−6^ (binary) and 5 × 10^−7^ (three-class) for MST. All models were trained for up to 300 epochs, with early stopping based on the validation loss to prevent overfitting. The batch sizes ranged from 18 to 64, depending on the memory requirements of the models. All models were implemented in PyTorch 2.1.2 with CUDA 12.1 and trained on a single NVIDIA RTX A6000 GPU of 48 GB memory. Final performance was evaluated using standard classification metrics, including accuracy, precision, recall, F1 score, and the area under the receiver operating characteristic curve (AUC). Confusion matrices and ROC curves were generated to provide a detailed view of the class-wise prediction performance.

### 2.6 Interpretability analysis

To gain insight into each model’s decision-making process and assess whether its predictions were grounded in clinically relevant features, we employed saliency-based visualization methods specific to each model architecture. For CNNs, we used gradient-weighted class activation mapping++ (Grad-CAM++) (30), which highlights spatially important regions by backpropagating gradients from the predicted output to convolutional feature maps. Grad-CAM++ was implemented using the MedCAM framework (31), targeting the final convolutional layer. For the MST model, self-attention maps were extracted from the final transformer layers. We utilized attention weights corresponding to the class token, averaged across all heads, to derive both the slice-level and in-plane saliency. The attention weights were then interpolated to the original image resolution for visualization. These transformer-derived saliency maps provided complementary interpretability to Grad-CAM++ by revealing the model’s attention across both anatomical slices and within-slice features. All saliency maps, including the Grad-CAM++ and transformer attention outputs, were generated for representative test samples and subsequently normalized and visualized using Matplotlib in Python. Additionally, to examine how much each arterial region contributes to model prediction, we segmented the internal carotid artery (ICA) and external carotid artery (ECA) from the MRA volumes using ITK-SNAP, and quantified relative saliency values within each arterial region for different WMH grades. The relative saliency value was obtained by dividing a mean value whithin each region by a global mean across the entire volume.

To determine the regional contributions of the input features to the model predictions, we performed occlusion sensitivity analysis. A sliding occlusion cube of size 8 × 8 × 8 voxels was systematically applied across the input volume with a fixed stride. At each position, the occluded input was re-evaluated, and the resulting change in the predicted class probability was recorded. The changes in probability across all occlusion positions were subsequently compiled into a heatmap reflecting model’s sensitivity to local perturbations. These occlusion heatmaps were also normalized and visualized using Matplotlib.

## 3 Results

### 3.1 Prediction of WMH presence

**Figure 2:**
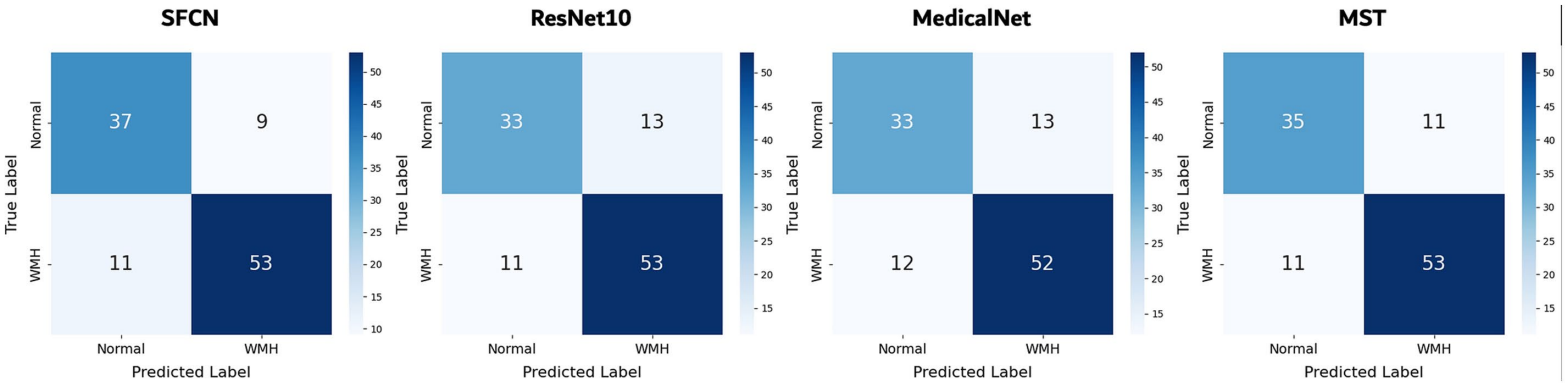
Confusion matrices of all models for classification of WMH presence. Results are shown for the best-performing seed of each model. Each cell indicates the number of test samples per ground truth and predicted class.

**Figure 3:**
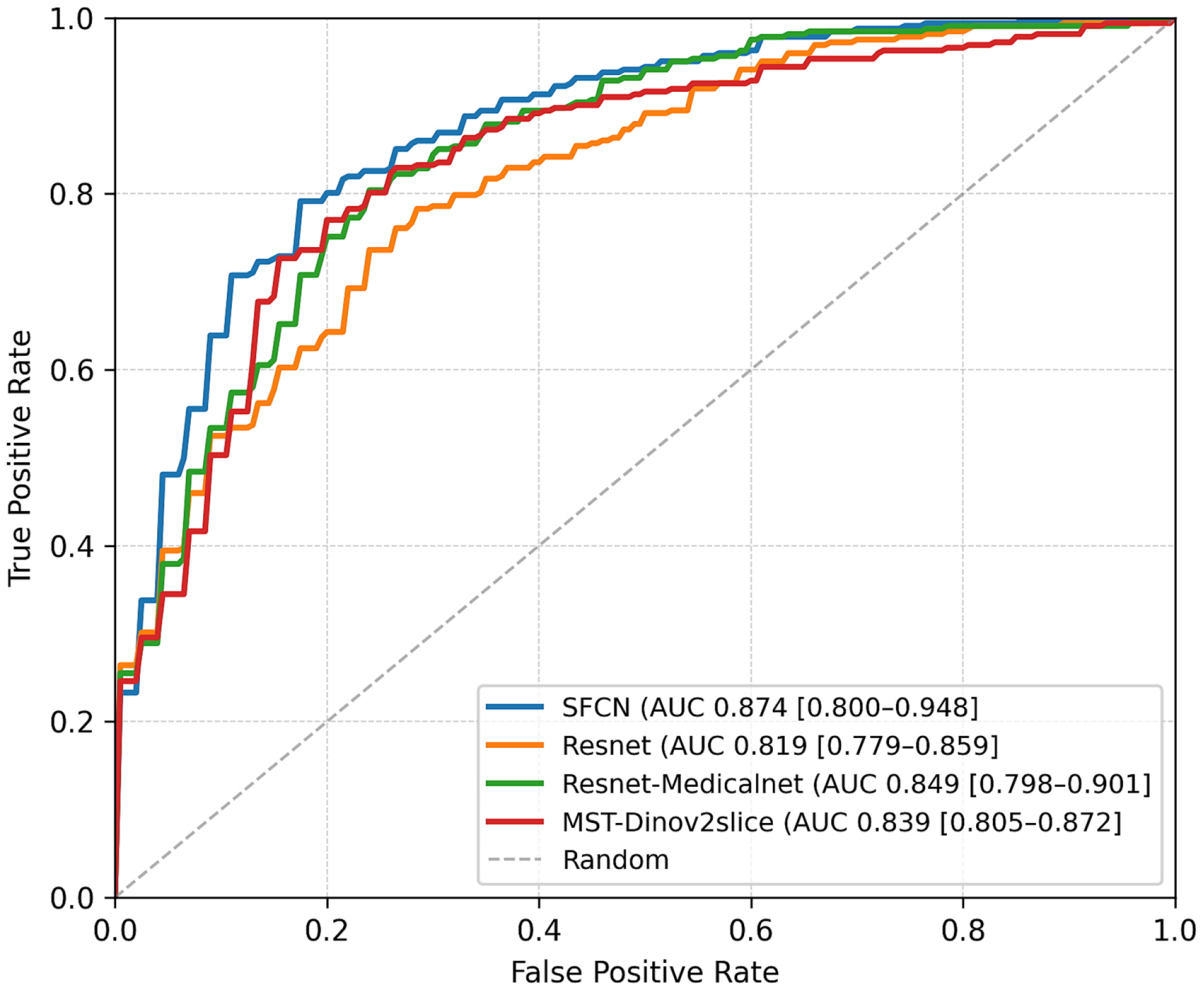
ROC curves and corresponding AUC values of all models for binary WMH classification. Results are presented for the best-performing seed of each model.

On the binary classification task, i.e., predicting the presence of WMH (grade 0 vs. grades 1–3), the SFCN model demonstrated the highest performance across all evaluation metrics. As summarized in Table 1, SFCN achieved an accuracy of 76.5%, recall of 0.765, and an AUC of 0.874 (averaged over multiple seeds), indicating strong classification capability with well-calibrated probabilistic predictions. The MST model exhibited comparable accuracy (76.5%) and recall (0.765), and achieved a slightly higher F1-score (0.764), although its AUC (0.839) was lower than that of SFCN. Among the remaining two models, MedicalNet demonstrated higher performance than ResNet10 across all reported metrics. The confusion matrices for all models (Figure 2) indicate balanced classification between normal and WMH grades, and the ROC curves (Figure 3) corroborate these findings. These results collectively indicate that all tested models are viable for WMH detection, with SFCN offering the most robust and generalizable performance.

**Table 1:** Quantitative performance metrics for prediction of WMH presence on test set.

Models	Metrics (mean, 95% CI)					Accuracy	Precision	Recall	F1	AUC
SFCN	0.765 (0.716–0.814)	0.774 (0.728–0.820)	0.765 (0.716–0.814)	0.762 (0.711–0.814)	0.874 (0.800–0.948)
ResNet10	0.738 (0.687–0.789)	0.737 (0.683–0.791)	0.738 (0.687–0.789)	0.736 (0.683–0.789)	0.819 (0.779–0.859)
MedicalNet	0.761 (0.735–0.787)	0.765 (0.740–0.790)	0.761 (0.735–0.787)	0.761 (0.735–0.787)	0.849 (0.798–0.901)
MST	0.765 (0.708–0.823)	0.764 (0.706–0.822)	0.765 (0.708–0.823)	0.764 (0.706–0.822)	0.839 (0.805–0.872)

### 3.2 Prediction of WMH severity

**Figure 4:**
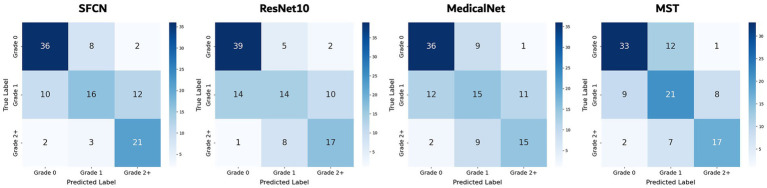
Confusion matrices of all models for WHM severity classification.

On the three-class WMH severity classification task (grade 0 vs. grade 1 vs. grade 2+), the SFCN model again achieved the best performance (accuracy of 63.5% and AUC of 0.827) as seen in Table 2. The MST model followed with an accuracy of 61.8% and an AUC of 0.824, demonstrating competitive performance, particularly in capturing severity differences. MedicalNet achieved higher accuracy (60.0%) than ResNet10 (57.9%), but its AUC (0.786) was lower than that of ResNet10 (0.810). Figure 4 presents the confusion matrices for all models. Most misclassifications occurred between adjacent grades, such as grade 1 being misclassified as either grade 0 or grade 2+, which reflects the gradual and continuous nature of WMH progression. Despite these challenges, SFCN demonstrated reasonable discriminative capability across all severity levels.

**Table 2:** Quantitative results for WMH severity classification.

Models	Metrics (mean, 95% CI)					Accuracy	Precision	Recall	F1	AUC
SFCN	0.635 (0.591–0.679)	0.634 (0.590–0.677)	0.635 (0.591–0.679)	0.629 (0.590–0.669)	0.827 (0.778–0.875)
ResNet10	0.579 (0.537–0.621)	0.574 (0.539–0.609)	0.579 (0.537–0.621)	0.572 (0.535–0.609)	0.810 (0.693–0.928)
MedicalNet	0.600 (0.574–0.627)	0.610 (0.586–0.634)	0.600 (0.574–0.627)	0.602 (0.577–0.627)	0.786 (0.752–0.819)
MST	0.618 (0.567–0.670)	0.627 (0.576–0.678)	0.618 (0.567–0.670)	0.620 (0.567–0.670)	0.824 (0.758–0.890)

The AUC values represent macro-averaged scores, computed as the unweighted means of the per-class AUCs based on a one-vs.-rest scheme.

### 3.3 Saliency map

**Figure 5:**
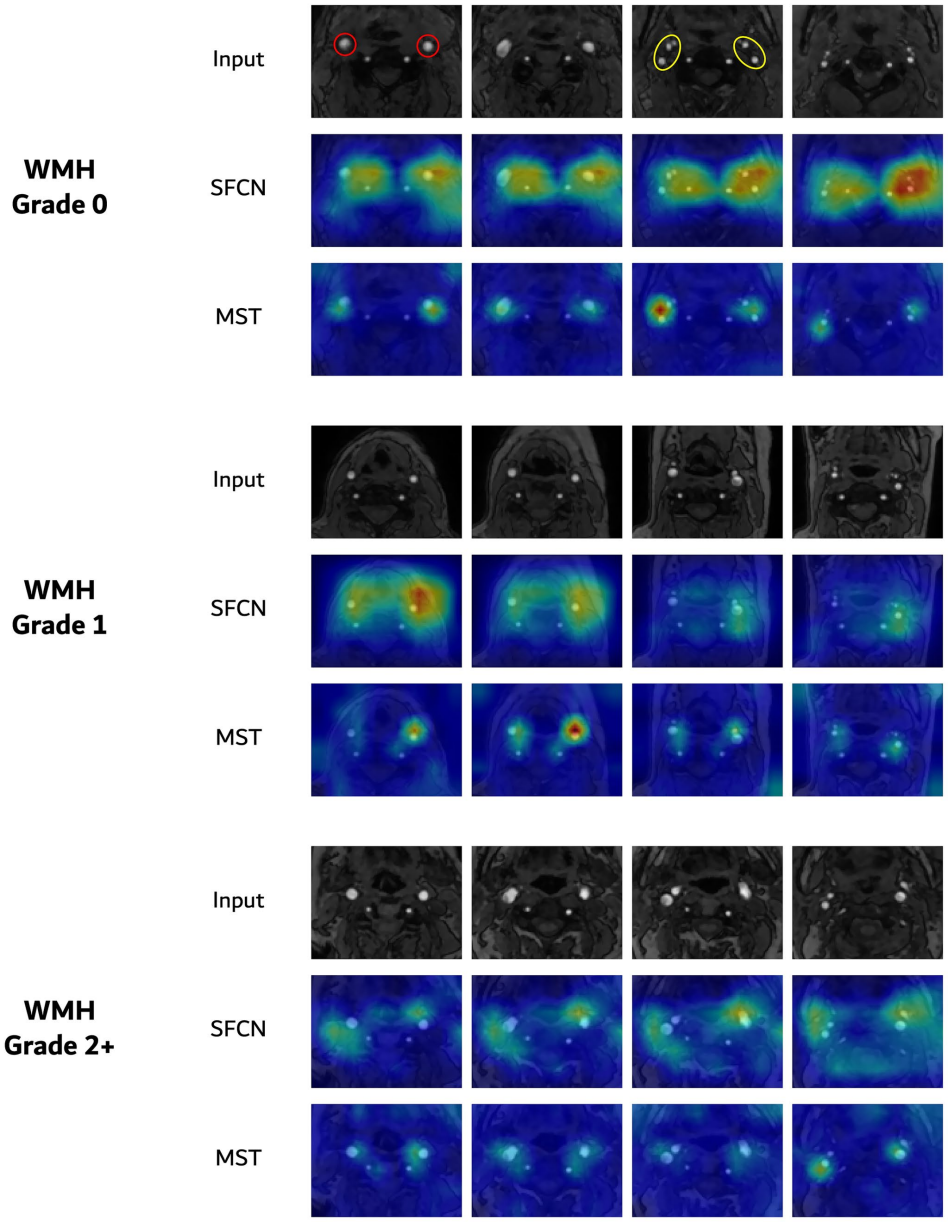
Saliency visualizations for three-class WMH severity classification. In each group, the top row presents axial slices of TOF MRA, followed by the corresponding saliency maps from the SFCN and MST models. The red and yellow circles for grade 0 indicate the common carotid arteries and the internal/external carotid arteries, respectively.

Figure 5 shows the saliency maps for the two representative architectures—SFCN and MST— that achieved the highest performance in the WMH severity classification task. For each model, the saliency maps were generated from the same subject across the three WMH severity grades (grade 0, 1, and 2+). SFCN and MST highlighted similar anatomical regions despite the substantial differences between their architectures (based on convolution and self-attention operations, respectively). Across severity levels, both models consistently highlighted vascular regions, with particularly strong activation around the carotid bifurcation, including the common carotid artery and its division into the internal and external carotid arteries. For a representative test case, quantitative analysis of volume-normalized activation densities from both models showed ICA/ECA density ratios ranging from about 1.3 to 3.5, indicating a consistently greater focus on the ICA than on the ECA. Apart from supporting the hypothesis that carotid MRA contains vascular features that may be informative for WMH assessment, these observations underscore the interpretability of both CNN-based and transformer-based deep learning models in this context.

### 3.4 Occlusion sensitivity

**Figure 6:**
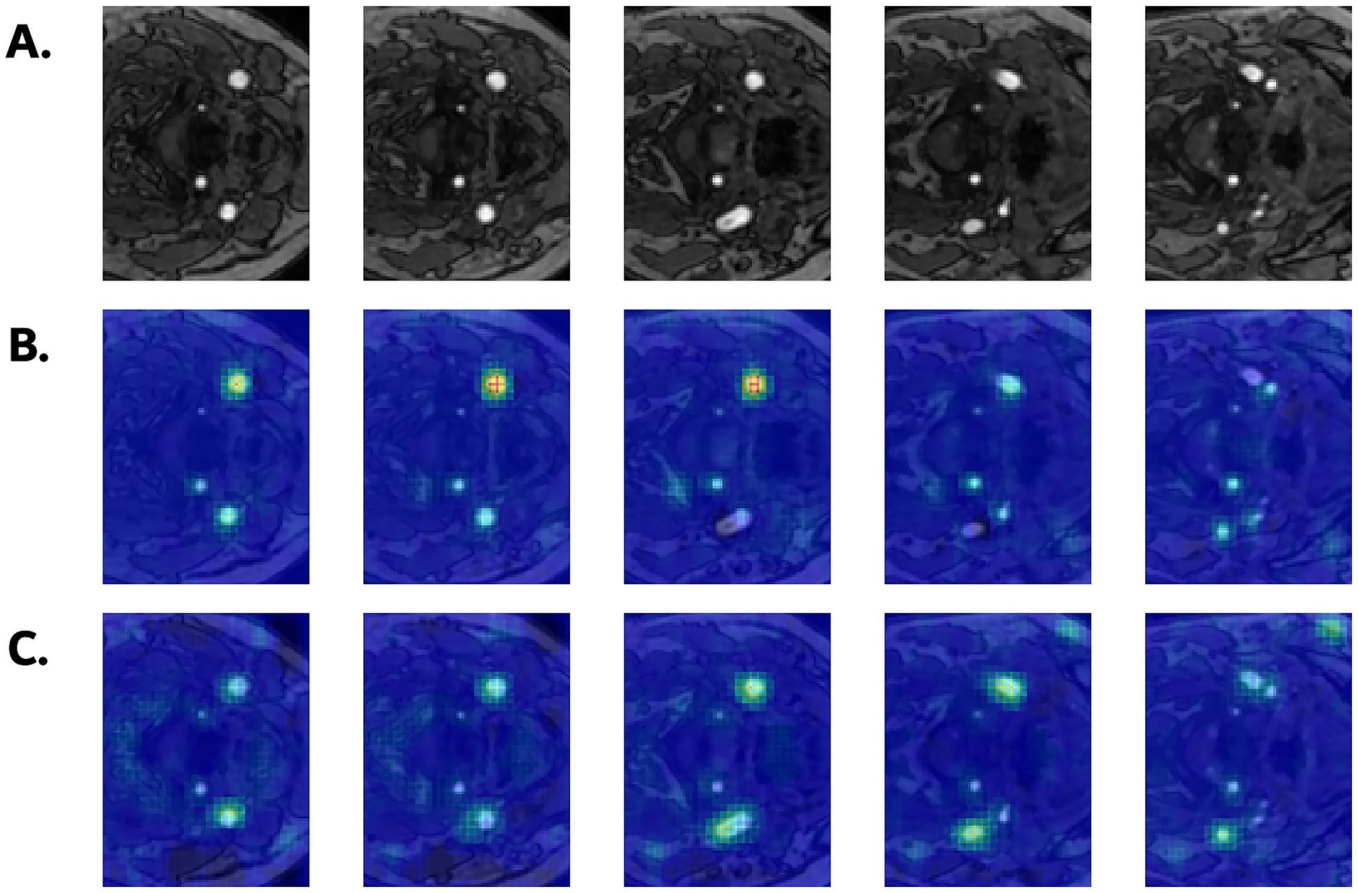
**(A)** Axial input slices of original TOF MRA from a subject with WMH (grade 2). Corresponding occlusion sensitivity maps from **(B)** SFCN and **(C)** MST models. Warmer regions indicate larger drops in predicted WMH probability.

Figure 6 illustrates the occlusion sensitivity maps for the two representative models. In subjects with WMH, occluding voxels corresponding to the carotid arteries consistently led to substantial drops in the predicted WMH probability, which suggests that the models place considerable emphasis on these vascular regions during classification. This effect was particularly pronounced in SFCN, which consistently highlighted the carotid artery along the slice axis, with high intensities around the carotid bifurcation. MST showed smaller changes overall, possibly due to its slice-wise attention mechanism; nevertheless, the most affected regions were still located around the carotid arteries. Together with the saliency maps, the occlusion sensitivity maps prove that both models attend to carotid vascular structures for WMH prediction.

## 4 Discussion

We investigated the feasibility of using carotid TOF MRA images to predict WMH with a deep learning-based framework. While previous studies have primarily reported statistical associations between carotid morphological features—such as vessel diameter and tortuosity—and WMH burden (14, 16), our focus was on direct image-based prediction. To the best of our knowledge, this study represents the first attempt to directly leverage raw carotid MRA images for WMH classification in an end-to-end manner.

To explore how WMH prediction can be achieved from carotid imaging, we designed two classification tasks: binary classification for WMH presence and three-class classification for WMH severity. In the binary task, all models—SFCN, ResNet10, MedicalNet, and MST—demonstrated comparable performance, suggesting that carotid TOF MRA provides sufficient information to detect the presence of WMH. Among the tested models, SFCN performed the best, achieving an AUC of 0.882 and a recall of 0.816. In the more challenging three-class severity classification task, SFCN again achieved the highest performance, exhibiting a macro-averaged AUC of 0.847 and a recall of 0.670, followed by the transformer-based MST model. These results indicate that both convolutional and transformer architectures can capture clinically relevant features for WMH severity prediction. Interpretability analyses based on saliency mapping and occlusion sensitivity suggested that the models consistently attended to carotid artery regions. This pattern concurs with previous reports of associations between carotid vascular characteristics and WMH burden. SFCN and MST both showed prominent attention to the carotid artery, including the bifurcation, which suggests that vascular features captured in TOF MRA may serve as informative imaging markers for cSVD. This implies that extracranial carotid vessels could reflect intracranial microvascular pathology, thus offering a non-invasive surrogate for brain health.

WMH burden demonstrates complex associations with cervical carotid artery imaging findings through interconnected morphological and hemodynamic pathways. Morphologically, abnormal arterial tortuosity correlates with severe WMH by creating disturbed flow patterns that increase endothelial shear stress and promote microvascular dysfunction (16). From a hemodynamic perspective, carotid diameter enlargement and enhanced pulsatile flow transmission expose small cerebral vessels to increased pressure fluctuations, contributing to periventricular WMH development by disrupting the blood–brain barrier (14). Although atherosclerosis further complicates this relationship (34), our study only included individuals without hemodynamically significant stenosis. In such cases, non-stenotic carotid plaques may still contribute to WMH burden through mechanisms such as microembolic events, impaired cerebrovascular reactivity, or systemic vascular risk factors including hypertension and aging. The multifactorial nature of these effects—encompassing subclinical flow disturbances, embolic potential, and shared risk profiles—underscores the inexplicability of WMH burden by cervical carotid artery morphology alone. Nevertheless, our results demonstrate that deep learning–based techniques can successfully predict the presence and severity of WMH from cervical carotid artery images, particularly in the absence of significant stenosis; this indicates that our models could learn vascular features predictive of WMH burden from vessel morphology. If our deep learning-based approach can identify vascular morphological changes affecting WMH burden even in patients without stenosis, it has the potential to enable the discovery of novel imaging biomarkers serving as independent WMH risk factors.

While carotid MRA commonly coexists with brain MRI, the predictive model adds value particularly in settings where only MRA is performed in certain clinical scenarios—such as follow-up of known carotid pathology, screening in high-risk but asymptomatic individuals, or institutional workflow constraints. In these situations, carotid MRA-based prediction could inform targeted recommendations for subsequent brain MRI, ensuring timely detection of clinically significant WMH in patients who might otherwise not undergo brain imaging. In addition, while this study focused on carotid MRA data, the underlying principles suggest strong potential for extending these predictive capabilities to cross-sectional imaging modalities such as neck CT or CT angiography, with appropriate adaptation and validation. This could broaden the reach of WMH risk assessment and improve diagnostic efficiency across a wider range of clinical scenarios.

WMHs are typically assessed on brain MRI through visual rating scales, such as the Fazekas scale, which grade lesions based on size and confluence (35). Although widely used, this qualitative method is prone to inter-rater variability. To address these limitations, various automated segmentation tools, such as Lesion Segmentation Tool (36) and BIANCA (37), have been developed to improve objectivity and reproducibility, which has enabled quantitative assessment. Recent advances in deep learning have further enhanced WMH segmentation. Models based on CNNs have demonstrated notable capabilities in segmenting WMHs (38–40). However, certain challenges remain, particularly in terms of the accuracy in detecting small and subtle WMHs and the robustness of domain adaptation across different scanners and imaging protocols (39, 41). Consequently, despite advances in automated segmentation, visual grading remains a widely used method for WMH assessment (42). Therefore, we assessed WMH severity using the widely used modified Fazekas scale, achieving almost perfect inter-rater reliability (weighted kappa of 0.879).

Unlike traditional WMH assessment methods, our deep learning–based framework enables the prediction of WMH severity directly from carotid TOF MRA in a fully automated fashion. By removing the dependence on WMH lesion annotations and brain imaging availability, this approach can reduce processing time and costs while enabling risk assessment in settings where only carotid imaging is performed. In addition, it may capture subclinical carotid features linked to WMH pathophysiology beyond human-observable morphological metrics, supporting earlier, non-invasive screening and potentially revealing new imaging biomarkers.

Despite our significant findings from this study, several limitations must be acknowledged. First, the progressive and continuous nature of WMH presents an inherent challenge in discrete classification, particularly under intermediate severity (e.g., grade 1), for which the models performed relatively poorly. Second, due to limited data on advanced cases, grades 2 and 3 had to be combined, which potentially reduced the classification granularity. Third, our study utilized single-center dataset acquired with single scanner, which limits applicability to datasets acquired under different hardware settings or protocols, and therefore external validation is essential to demonstrate generalizability. Furthermore, no clinical validation was performed against clinical outcomes such as stroke risk or cognitive scores. This limits the ability to fully assess the clinical utility of the proposed method. In future work, we plan to incorporate such clinical variables to enable quantitative validation and strengthen clinical utility. Studies involving multi-center data and larger and more diverse patient cohorts, particularly those with advanced WMH severity, will also be essential for improving model robustness and enabling finer-grained classification. To ensure applicability across different scanners and acquisition protocols, we will explore preprocessing and adaptation strategies, including MRI harmonization to standardize intensity scales and align feature representations, together with domain adaptation techniques to reduce inter-scanner variability (43, 44). Integrating these approaches with multi-center validation will help achieve robust, generalizable performance and support consistent WMH risk assessment across varied clinical settings.

## 5 Conclusion

This study introduced a deep learning-based approach for predicting WMH in the brain using carotid TOF MRA as the sole imaging modality. Our findings demonstrate that both convolutional and transformer-based models can effectively extract vascular image features relevant to WMH detection and grading, without any reliance on brain MRI. Attention patterns observed in saliency-based interpretability analyses corroborate the association between carotid structure and WMH burden. The proposed framework provides WMH predictions based solely on extracranial carotid imaging, serving a practical basis for cerebrovascular risk assessment. Furthermore, it may offer complementary value alongside conventional brain MRI by capturing vascular morphological features associated with WMH burden, even without significant stenosis.

## Data Availability

The original contributions presented in the study are included in the article/supplementary material, further inquiries can be directed to the corresponding author/s.
